# Contributions of Social Factors to Disparities in Prostate Cancer Risk Profiles among Black Men and Non-Hispanic White Men with Prostate Cancer in California

**DOI:** 10.1158/1055-9965.EPI-21-0697

**Published:** 2021-11-30

**Authors:** David J. Press, Salma Shariff-Marco, Daphne Y. Lichtensztajn, Diane Lauderdale, Adam B. Murphy, Pushkar P. Inamdar, Mindy C. DeRouen, Ann S. Hamilton, Juan Yang, Katherine Lin, Donald Hedeker, Christopher A. Haiman, Iona Cheng, Scarlett Lin Gomez

**Affiliations:** 1 Department of Public Health Sciences, University of Chicago, Chicago, Illinois.; 2 Department of Preventive Medicine, Northwestern University Feinberg School of Medicine, Chicago Illinois.; 3 The Center for Health Information Partnerships (CHiP), Institute of Public Health and Medicine, Northwestern University Feinberg School of Medicine, Chicago, Illinois.; 4 Greater Bay Area Cancer Registry, Department of Epidemiology and Biostatistics, University of California, San Francisco, School of Medicine, San Francisco, California.; 5 Department of Epidemiology and Biostatistics, University of California, San Francisco, School of Medicine, San Francisco, California.; 6 Helen Diller Family Comprehensive Cancer Center, San Francisco, California.; 7 Department of Urology, Northwestern University Feinberg School of Medicine, Chicago, Illinois.; 8 Department of Preventive Medicine, Keck School of Medicine, University of Southern California (USC), Los Angeles, California.

## Abstract

**Background:**

Black men are more likely than Non-Hispanic White (NHW) men to be diagnosed with high-risk prostate cancer. We examined the extent to which social factors were associated with differences in prostate cancer risk profiles between Black men and NHW men [using a modification to the original D'Amico risk groups based on prostate specific antigen (PSA), Gleason score (GS), and TNM stage (stage)], based on individual and combined clinicopathologic characteristics.

**Methods:**

We conducted a cross-sectional population-based study of 23,555 Black men and 146,889 NHW men diagnosed with prostate cancer in the California Cancer Registry from 2004 to 2017. We conducted multivariable logistic regression to examine the association of year of diagnosis, block group-level neighborhood socioeconomic status (nSES), marital status, and insurance type on differences in prostate cancer risk profiles between Black and NHW men.

**Results:**

High PSA (>20 ng/mL), GS, stage, individually and combined prostate cancer risk profiles were more common among Black men versus NHW men. In fully adjusted models, relative to NHW men, we observed a persistent 67% increased odds of high PSA among Black men. nSES was the factor most strongly associated with racial disparity in high PSA, accounting for 25% of the difference. Marital status was the factor that was second most associated with a racial disparity.

**Conclusions:**

nSES was the factor most strongly associated with racial disparities in high PSA prostate cancer.

**Impact:**

The influence of nSES on racial disparities in PSA, GS, stage, and prostate cancer risk profiles warrants further consideration.

## Introduction

Prostate cancer is the most common cancer among men in the United States. In 2021, Black men are projected to experience 1.8 times the incidence and 2.1 times the mortality as non-Hispanic White (NHW) men (1). Prostate-specific antigen (PSA), Gleason score (GS), and stage are prognostic factors of prostate cancer, which together can be used to indicate prostate cancer risk profile for clinical decision-making (2). Black men with prostate cancer present with higher PSA levels on average compared with other racial groups, and for a given level of PSA, Black men have larger tumor volumes than NHW men (3–6). In California, we previously reported a 60% higher age-adjusted prostate cancer mortality among Black men relative to NHW men, which was reduced to the null after adjustment for tumor, sociodemographic, institutional, and neighborhood characteristics (7). However, it remains unclear what factors contribute to the higher risk of advanced prostate cancer at diagnosis among Black men.

Social determinants of disparities in cancer outcomes (e.g., clinicopathologic presentation, diagnosis, treatment, and survival; ref. 8) are complex and intersecting. What we observe as racial disparities—adverse health consequences of racism for historically marginalized racial groups—may partially reflect overlapping inequities across other social factors associated with cancer outcomes (7, 9–13). Individuals who reside in resource-poor settings as measured by low neighborhood socioeconomic status (nSES) are more likely to experience social isolation, stressors, have reduced access to medical and social services (14–22) and also experience disparate prostate cancer outcomes (2, 7, 23). In addition, patient-level social factors including health insurance status type and marital status are associated with disease stage and mortality (7, 24). Determining the relative contribution of intersecting social factors (at the individual- and contextual-level) to prostate cancer risk is an area of ongoing investigation (2, 7, 23, 25). To our knowledge, only one study has evaluated associations between these factors (in addition to race) and risk of advanced prostate cancer at diagnosis (2). Elucidating these factors may provide insight to mitigate the Black-White racial disparity in prostate cancer survival. To further examine intersecting social factors that may impact prostate cancer risk profile, we conducted a population-based study using California Cancer Registry (CCR) data of Black men and NHW men in California with prostate cancer from 2004 to 2017 with detailed information on PSA, GS, and stage, in addition to information on individual characteristics (age at and year of diagnosis, marital status, insurance status) and nSES.

## Materials and Methods

### Study population

From the CCR, we identified 25,886 Black men and 160,897 NHW men residing in California diagnosed with first primary invasive prostate cancer [International Classification of Disease for Oncology, 3rd edition [ICD-O-3] site code C619; ref. 26] during the period January 1, 2004 to December 31, 2017. The population-based CCR comprises three regional registries that are a part of the National Cancer Institute's Surveillance, Epidemiology, and End Results (SEER) program, which maintains the highest level of registry data quality and accuracy. We limited the earliest year of diagnosis to 2004 since PSA and GS were incomplete in cancer registry data before 2004 (27). We limited the study to men with pathologically confirmed adenocarcinoma or other prostate cancer histology (ICD-O-3 morphology codes 8000–8110, 8140–8576, 8940–8950, and 8980–8981; ref. 26). The final cross-sectional study population included 23,555 Black men and 146,889 NHW men; however, slightly different numbers of cases were included for each outcome studied on the basis of data availability. Specifically, the study samples with complete data for each prostate cancer risk profile outcome included: PSA data for 21,643 Black men and 131,755 NHW men; GS data for 22,255 Black men and 138,393 NHW men; clinical stage data for 22,854 Black men and 142,487 NHW men; and combined risk profile data for 21,658 Black men and 132,050 NHW men. A flow diagram is presented in Supplementary Fig. S1.

### Variables

#### Independent variables

Race/ethnicity was classified as NHW for White men who were also non-Hispanic and Black for Black/African American men who were either Hispanic or non-Hispanic. Insurance type was defined as primary payer (no insurance, private, Medicare only, any public/Medicaid/military, and unknown or missing) based on the last report received by the cancer registry for a given diagnosis. nSES was measured using a previously defined composite index score developed by principal components analyses of 2000 Census (for diagnoses 2004–2005) or 2008 to 2012 American Community Survey (for diagnoses 2006–2017) data on education, occupation, employment, household income, poverty, and rent and house values (28,29) linked to the census block group. Address of residence at time of diagnosis was geocoded and used to assign a census block group. Each cancer case was assigned to a census block group nSES quintile based on the statewide distribution of nSES scores, separately derived for 2000 and 2010. Registry information on individual characteristics (age and year of diagnosis, marital status, insurance type based on primary payer, and residence at diagnosis) were abstracted from the medical record.

#### Outcome variables

We used CCR data items on PSA, GS, and American Joint Committee on Cancer stage (27) to categorize men into “low,” “intermediate,” and “high” prostate cancer risk groups based on a modification of D’Amico risk groups (30) and National Comprehensive Cancer Network (NCCN) risk categories (31), using stage, GS, and PSA. Low included low-risk (N0 and M0 and T1/T2a and Gleason ≤6 and PSA <10 ng/mL) and intermediate-risk (N0 and M0 and T2b/T2c or biopsy Gleason 7 or PSA 10–20 ng/mL); and high included high-risk (T3/T4 or Gleason 8+ or PSA >20 ng/mL or N1 or M1). For primary analyses, the overall combined risk group was dichotomized between low and high, with the intermediate group combined with the low group. In secondary analyses, we also examined risk stratification within each of the risk group component measures based on three categories within each component: PSA <10 ng/mL (low), 10–20 ng/mL (intermediate), >20 ng/mL (high); GS <7 (low), 7 (intermediate), ≥8 (high); and stage T1/T2a (low); T2b/T2c (intermediate); T3/T4 or N1 or M1 (high).

### Statistical analyses

Descriptive analyses of prostate cancer prognostic factors and patient characteristics by race were assessed by comparing frequencies and percent. Chi-squared tests were performed, but considered of limited practical utility due to the large sample size. To examine racial disparities in risk of advanced prostate cancer for each prostate cancer prognostic factor (i.e., PSA, stage, GS, and combined risk), we used multivariable logistic regression to estimate ORs and 95% confidence intervals (CI) across race, with NHW as the reference and each prostate cancer prognostic factor as the outcome in separate models. Classifications of prostate cancer prognostic factors were analyzed as binary variables with low and intermediate categories combined. Each multivariable model was sequentially adjusted for age at diagnosis, year of diagnosis, marital status, insurance type, and nSES. To examine whether observed differences in Black-NHW ORs were independent of other prognostic factors included in the overall combined prostate cancer risk profile, we developed a series of models, as follows:

- Binary PSA as the outcome (≤20 vs. >20). Fully adjusted models were stratified by binary GS (≤7 vs. ≥8) and binary stage (T1/T2a, T2b/T2c vs. T3/T4 or N1 or M1).
- Binary GS as the outcome. Fully adjusted models were stratified by binary PSA and binary stage.
- Binary stage was the outcome. Fully adjusted models were stratified by binary PSA and binary GS.
- Binary combined risk as the outcome. These models were not stratified.

To assess potential differences within prostate cancer prognostic factor groupings, we also developed fully adjusted multinomial logistic regression models using the three risk group categories as in our prior research (2), using the low categories as the reference group.

To examine the relative influence of each covariable (i.e., age at diagnosis, year of diagnosis, insurance type, marital status, and nSES) on observed Black-NHW disparities in prostate cancer risk combined and separately we used a previously developed method (32). Briefly, the baseline model included race plus age. The prostate cancer risk disparity for a particular model was D\ = \ \sqrt {( {\sum {n_i}{{\{ {{\beta _i} - {{\bar{\beta }}_ \bullet }} \}}^2}} )/\sum {n_i}} $ , the sample-size weighted standard deviation of OR estimate for Black men relative to NHW men. Here, {\beta _i}$ is the {\mathrm{lo}}{{\mathrm{g}}_e}{\mathrm{OR}}$ estimate of Black men relative to NHW men, {n_i}$ is the sample size of Black men, and {\bar{\beta }_ \bullet }$ is the sample size-weighted mean for {\beta _i}$ . The relative influence was then defined as ( {\{ {{D_ - } - {D_ + }} \}/{D_0}} ) \times 100\ $ in which {D_0}$ was the OR from the baseline model, {D_ - }$ was the OR from the model without the covariable of interest, and {D_ + }$ was the OR from the model with the covariable of interest. In the multivariable context, {D_ - }$ was the OR from the model with the covariable of interest, and {D_ + }$ was the OR from the model without the covariable of interest. The influence of each covariable on Black-NHW disparities first was tested in a base model to identify univariable influence: race plus age plus covariable. Covariables were then ranked in order of their univariable influence on prostate cancer risk disparities (i.e., by how much the logistic regression OR predicting prostate cancer risk decreased when included in the base model), sequentially added to the baseline model by the univariable influence rank order, sequentially assessing the change in OR as a measure of the relative change in Black-NHW disparity (i.e., the proportion of the total disparity contributed by that covariable, after accounting for previously added covariables). We also obtained a measure of multivariable influence comparing the baseline model and the multivariable models including all covariables except for the covariable of interest. The process was performed separately for each prognostic factor of prostate cancer risk profile outcome. To check the robustness of the findings, we used the approach described in Gelman 2008 (33) to standardize covariates, and found that the results did not change using this approach; thus, we only present the first set of results.

To examine the possibility that ORs were an overestimate of risk ratios (RR), we calculated RRs and compared them with ORs, using the equation: {\mathrm{RR}} = {\ \frac{{{\mathrm{OR}}}}{{( {1 - {\mathrm{pre}}{{\mathrm{v}}_0}} ) + ( {{\mathrm{OR}} \times {\mathrm{pre}}{{\mathrm{v}}_0}} )}}$ , where {\mathrm{pre}}{{\mathrm{v}}_0}$ is the prevalence of the outcome among NHW men. In addition, we generated multiple imputations of missing covariable data, re-ran the multinomial regression analyses with these imputed values, and compared our models with and without multiple imputations to assess whether results differed between models with and without imputed values for missing covariables. In addition, we imputed missing outcome data based on covariables in the model using discriminant function method (https://documentation.sas.com/doc/en/pgmsascdc/9.4_3.4/statug/statug_mi_ details09.htm). Given that the maximum percentage of missing outcome is 10%, we generated 10 multiple imputation samples to achieve 99% efficiency. We re-ran multinomial regression analyses with these imputed data, and the OR estimates from models with and without multiple imputations were compared with assess whether results differed between models with and without missing values in outcome variables. We did not perform multiple comparisons tests.

This study was based on de-identified cancer registry data collected as part of the California statewide cancer registry reporting mandate. The analyses is approved for human subjects research through the Greater Bay Area Cancer Registry Institutional Review Board protocol at the University of California. All statistical comparisons were two-sided. We used SAS 9.4 for multivariable logistic regression analyses.

## Results

### Descriptive characteristics

Descriptive characteristics for the 23,555 Black men and 146,889 NHW men diagnosed with prostate cancer from 2004 to 2017 are presented in **Table 1**. The age range of the cohort was 21 to 102 years. Black men with prostate cancer had higher proportions of high risk prostate cancer categories than NHW men (i.e., within the components of PSA, GS, stage, as well as for combined risk). Differences were greatest for high PSA (>20 ng/mL; Black men: 16.3%; NHW men: 9.8%). Black men were more often diagnosed at younger age (<55 years) compared with White men (16.2% and 8.6% respectively). In comparison to NWH men, Black men were more likely to reside in lowest SES neighborhoods (24.2% compared with 6.6%), more likely to be unmarried (41.7% compared with 24.2%), less likely to have Medicare insurance (13.2% compared with 23.7%), and more likely to have public insurance (28.9% compared with 19.1%). All frequencies examined {\chi ^2}$ p-values < 0.001. Higher proportions of worse prognosis for each factor were observed for Black men relative to NHW men, for older men relative to younger men, for widowed men, for men residing in lower SES neighborhoods, and for men with unknown or missing insurance status. Supplementary Table S1 provides characteristics of men with missing prostate cancer outcome data by age. We observed some indication that data were missing not at random.

**Table 1.** Patient demographic and prostate cancer (PCa) characteristics among 23,555 Black men and 146,889 NHW men diagnosed with first primary invasive prostate cancer from 2004 to 2017 in California.

	Race				
	NHW men		Black men		
Patient and PCa characteristics	*n*	Col (%)	*n*	Col (%)	Total
PCa prognostic factor^a^					
Prostate-specific antigen (PSA) category					
Low (<10 ng/mL)	96,042	65.3	13,669	58.0	109,711
Intermediate (10 to <20 ng/mL)	21,283	14.4	4,139	17.6	25,422
High (20+ ng/mL)	14,430	9.8	3,835	16.3	18,265
Missing	15,134	10.3	1,912	8.1	17,046
Gleason score (GS) risk category					
Low (<7)	57,430	39.1	8,648	36.7	66,078
Intermediate (7)	57,581	39.2	9,689	41.1	67,270
High (8+)	23,382	15.9	3,918	16.6	27,300
Missing	8,496	5.8	1,300	5.5	9,796
Stage risk category					
Low (N0, M0, and T1/T2a)	89,814	61.1	15,068	63.9	104,882
Intermediate (N0, M0, and T2b/T2c)	35,143	23.9	4,615	19.6	39,758
High (N1, M1, and/ or T3a+)	17,443	11.9	3,161	13.4	20,604
Missing	4,489	3.1	711	3.0	5,200
Combined National Comprehensive Cancer Network (NCCN) PCa risk category					
Low	34,245	23.3	5,233	22.2	39,478
Intermediate	63,265	43.1	9,862	41.9	73,127
High	34,540	23.5	6,563	27.9	41,103
Missing	14,839	10.1	1,897	8.1	16,736
Individual characteristics					
Age at diagnosis (years)					
<55	12,555	8.6	3,818	16.2	16,373
55–64	48,011	32.7	9,288	39.4	57,299
65–74	57,038	38.8	7,681	32.6	64,719
75+	29,285	19.9	2,768	11.8	32,053
Year of diagnosis					
2004	12,008	8.2	1,664	7.1	13,672
2005	10,632	7.2	1,607	6.8	12,239
2006	12,056	8.2	1,809	7.7	13,865
2007	12,903	8.8	1,930	8.2	14,833
2008	11,964	8.1	1,811	7.7	13,775
2009	11,556	7.9	1,870	7.9	13,426
2010	11,350	7.7	1,893	8.0	13,243
2011	11,485	7.8	1,837	7.8	13,322
2012	9,499	6.5	1,729	7.3	11,228
2013	9,138	6.2	1,637	7.0	10,775
2014	8,328	5.7	1,418	6.0	9,746
2015	8,605	5.9	1,431	6.1	10,036
2016	8,580	5.8	1,414	6.0	9,994
2017	8,785	6.0	1,505	6.4	10,290
Marital status					
Single, never married^b^	15,538	10.6	5,059	21.5	20,597
Married	100,470	68.4	12,170	51.7	112,640
Separated	1,032	0.7	474	2.0	1,506
Divorced	9,621	6.6	2,242	9.5	11,863
Widowed	5,830	4.0	939	4.0	6,769
Unknown	14,398	9.8	2,671	11.3	17,069
Insurance type^c^					
No insurance	1,139	0.8	336	1.4	1,475
Private	76,285	51.9	12,500	53.1	88,785
Medicare only	34,767	23.7	3,100	13.2	37,867
Any public/medicaid/military	28,116	19.1	6,795	28.9	34,911
Unknown or missing	6,582	4.5	824	3.5	7,406
Neighborhood characteristics					
nSES at diagnosis					
Quintile 1 (low)	9,619	6.6	5,689	24.2	15,308
Q2	19,776	13.5	5,540	23.5	25,316
Q3	29,076	19.8	4,957	21.0	34,033
Q4	37,767	25.7	4,538	19.3	42,305
Quintile 5 (high)	50,651	34.5	2,831	12.0	53,482
Total	146,889	100.0	23,555	100.0	170,444

Note: All {\chi ^2}$*P* values < 0.001.

^a^PCa risk profile stratification criteria based on the NCCN classification using TNM stage, GS, and PSA level. Low included low-risk (T1/T2a and GS ≤ 6 and PSA <10 ng/mL) and intermediate-risk (N0 and M0 and T2b/T2c or biopsy GS 7 or PSA 10–20 ng/mL); and high included high-risk (T3/T4 or GS 8+ or PSA >20 ng/mL or N1 or metastatic M1).

^b^Single, never married included unmarried or domestic partner (same sex or opposite sex, registered or unregistered other than common law marriage).

^c^Primary payer at diagnosis.

### Black-NHW disparities in prostate cancer risk profiles at diagnosis

**Figure 1.**
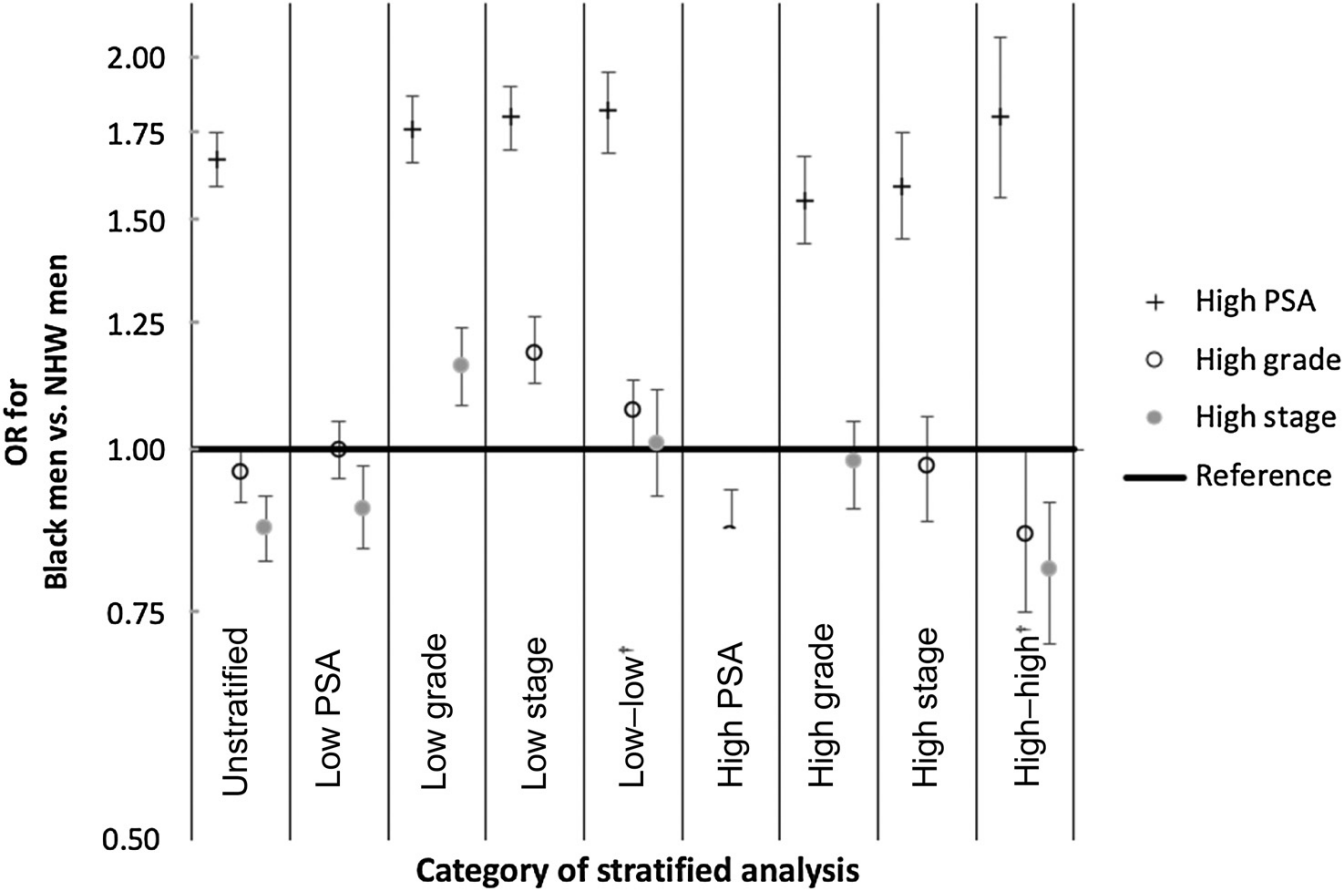
OR of PCa risk profiles for Black men relative to NHW men among men diagnosed with PCa from 2004 to 2017 in California using three risk categorizations (PSA,^a^ GS,^b^ and stage^c^), fully adjusted models series of stratified models defined by PSA^a^, GS, and stage^c^. All models adjusted for age at diagnosis, year of diagnosis, marital status, insurance type, and nSES. Unstratified model for outcome of PSA additionally adjusted for GS and stage (low and high). Unstratified model for outcome of GS additionally adjusted for PSA and stage (low and high). Unstratified model for outcome of stage additionally adjusted for PSA and GS (low and high). †, Low–low and high–high represent the strata for which the outcome is not modeled [i.e., for high PSA: low stage and low GS (low–low) and high stage and high GS (high–high); for high GS: low PSA and low stage (low–low) and high PSA and high stage (high–high); and for high stage: low PSA and low GS (low–low) and high PSA and high GS (high–high)]. ^a^PSA risk category low (≤20 ng/mL) vs. high (>20 ng/ mL). ^b^GS risk category low (<8) vs. high (8+). ^c^Stage risk categories low (N0, M0, and <T2b) and high (N1, M1, and/ or T3a+).

Figure 1 and Supplementary Table S2 provide results from multivariable models for the association between all covariables and each of the binary prostate cancer risk outcomes assessed. Black men had increased odds of high PSA, GS, stage, and combined risk, compared with NHW men (65%, 13%, 12%, and 27% increases, respectively). Further adjusting for GS and stage had no impact on the increased odds of high PSA among Black men relative to NHW men (OR, 1.67; 95% CI, 1.59–1.75; *P* < 0.001). However, the increased odds of high GS and high stage among Black men was attenuated to the null and beyond the null, respectively, when we include all specific risk measures in a single model. In these models, high PSA was strongly associated with high GS and high stage tumors; the OR for the association of PSA with GS was 6.27 (95% CI, 6.04–6.50; *P* < 0.001) in the model with high GS as the outcome; and the OR for the association of PSA with stage was 7.41 (95% CI, 7.12–7.73; *P* < 0.001) in the model with high stage as the outcome.

Table 2 shows results of fully adjusted models of the racial disparity considering three categories of risk for each specific prostate cancer risk measure to assess the pattern of the racial disparity across the three categories; with low risk as the referent. The most prominent disparity was evident for PSA; compared with NHW men, Black men had 40% increased odds of intermediate versus low PSA (OR, 1.37; 95% CI, 1.29–1.45) and nearly twice the odds of high versus low PSA (OR, 1.96; 95% CI, 1.86–2.06), even with adjustment for GS and stage. This pattern was not observed for GS for which, compared with NHW men, Black men had equivalent odds of high versus low GS after adjustment for PSA (OR, 1.00; 95% CI, 0.96–1.05). For stage, we observed inverse associations between intermediate versus low stage (OR, 0.78; 95% CI, 0.75–0.81) and high versus low stage (OR, 0.84; 95% CI, 0.80–0.88), suggesting that Black men were less likely than NHW men to present with intermediate or high stage disease after adjustment for PSA. Comparison of these results with those from the multiple imputations revealed <1% differences in ORs for all models. Black-NHW disparities in high PSA persisted across all stratified analyses; whereas attenuations to the null were observed for high GS and high stage when stratified by low PSA disease and beyond the null when stratified by high PSA (Fig. 1; Supplementary Table S3). We did not observe substantial differences in the OR and RR calculations (Supplementary Table S4).

**Table 2.** OR of PCa risk profiles among Black men relative to NHW men with outcomes of high PSA,^a^ GS,^b^ and stage^c^ disease among California residents with PCa from 2004 to 2017, by race.

	OR for Black men relative to NHW men				
	Intermediate vs. low		High vs. low		
PCa risk profile outcome modeled	OR	(95% CI)	OR	(95% CI)	*N* ^d^
PSA^a^	1.40	(1.35–1.46)	1.96	(1.86–2.06)	153,398
GS^b^	1.12	(1.08–1.15)	1.00	(0.96–1.05)	160,648
Stage^c^	0.78	(0.75–0.81)	0.84	(0.80–0.88)	165,244^e^
Combined National Comprehensive Cancer Network (NCCN) PCa risk category^f^	1.09	(1.05–1.13)	1.35	(1.29–1.41)	153,708

Note: All models adjusted for age at diagnosis (years), year of diagnosis, marital status, insurance type, and nSES. Model for outcome of PSA adjusted for GS (low, intermediate, high), and stage (low, intermediate, and high). Model for outcome of GS adjusted for PSA (low, intermediate, high). Model for outcome of stage adjusted for PSA (low, intermediate, high).

^a^PSA risk category low (<10 ng/mL), intermediate (10–20 ng/mL), and high (>20 ng/mL).

^b^GS risk category low (<7), intermediate (7), and high (8+).

^c^Stage risk categories low (N0, M0, and T1/T2a), intermediate (N0, M0, and T2b/T2c) and high (N1, M1, and/ or T3/T4).

^d^Models with PSA outcomes exclude men with missing data on PSA. Models with GS outcomes exclude men with missing data on PSA or GS. Models with outcomes of stage exclude men with missing data on PSA or stage.

^e^Excludes *n* = 97 (0.06%) for whom N stage and M stage are missing. These men were inferred to not have nodal involvement or metastases in binomial analyses.

^f^PCa risk stratification criteria based on the NCCN classification using TNM stage, GS, and PSA level. Low included low-risk (T1/T2a and GS ≤ 6 and PSA <10 ng/mL) and intermediate-risk (T2b/T2c or biopsy GS 7 or PSA 10–20 ng/mL); and high included high-risk (T3/T4 or GS 8+ or PSA >20 ng/mL or N1 or M1).

### Relative influence of covariables on Black–NHW disparities

**Figure 2.**
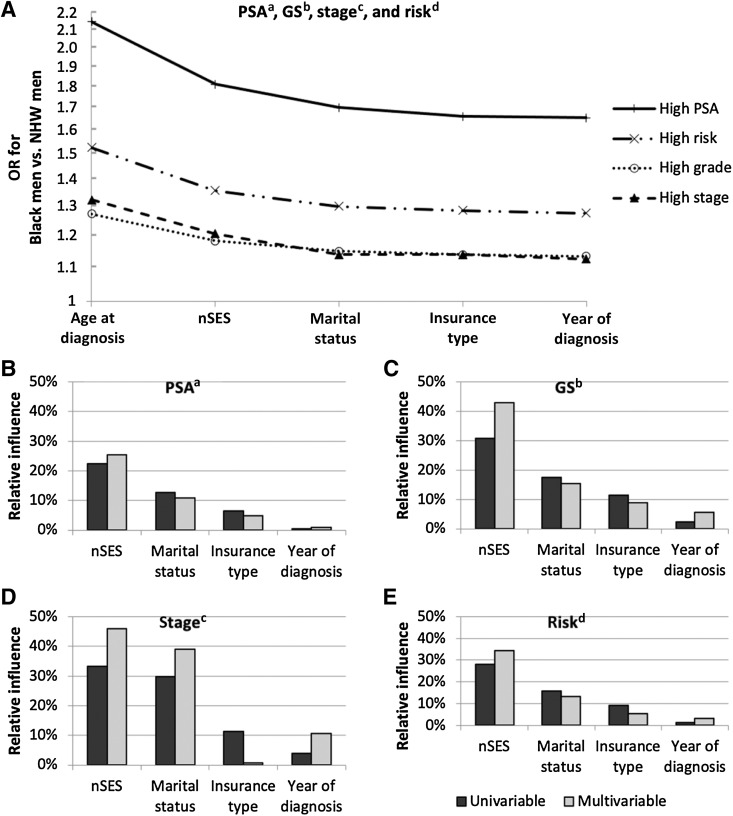
**A,** OR of PCa risk profiles (PSA,^a^ GS,^b^ stage,^c^ and combined risk^d^) for Black men compared with NHW men, for a sequence of logistic regression models, the leftmost of which includes racial/ethnic group alone adjusted for age at diagnosis, where variables are added in the order of their univariable significance, and where the rightmost represents the full baseline model. **B–E,** Univariable and multivariable relative influence of individual variables in the baseline model for prostate cancer risk profile outcomes. ^a^PSA risk category low (≤20 ng/mL) vs. high (>20 ng/mL). ^b^GS risk category low (<8) vs. high (8+). ^c^Stage risk categories low (N0, M0, and <T2b) and high (N1, M1, and/or T2b+). ^d^PCa risk stratification criteria based on the NCCN classification using TNM stage, GS, and PSA level. Low included low-risk (N0 and M0 and T1/T2a and GS ≤6 and PSA <10 ng/mL) and intermediate-risk (N0 and M0 and T2b/T2c or biopsy GS 7 or PSA 10–20 ng/mL), and high included high-risk (T3/T4 or GS 8+ or PSA >20 ng/mL or N1 or M1).

Results of models to assess relative influence of covariables on Black-NHW disparities in prostate cancer risk profiles are provided in Fig. 2. The age-adjusted odds of high versus low/intermediate PSA prostate cancer among Black men were 2.14 times those of NHW men (95% CI, 2.06–2.23). A large proportion of this racial disparity in PSA was attributable to nSES, which accounted for about 25.4% of the Black-NHW disparity in multivariable models. An additional 10.8% was explained by differences in marital status, and 4.9% was explained by differences in insurance status. Similarly, the largest proportion of the Black-NHW disparity in high GS, stage, and combined risk disease in multivariable models were attributable to differences in nSES, followed by marital status.

## Discussion

To examine the extent to which social factors contribute to racial disparities in prostate cancer risk profiles as defined by PSA, GS, clinical stage, and combined risk, we conducted a population-based study of all Black men and NHW men diagnosed with first primary invasive prostate cancer in California from 2004 to 2017. NSES was the most influential factor contributing to age-adjusted racial disparities in prostate cancer risk profile among Black men relative to NHW men for PSA, GS, stage, and combined risk; followed by marital status. Future studies are needed to elucidate the role of nSES and marital status in prostate cancer risk profile at diagnosis. Specific areas of interest include a careful consideration of what nSES may be measuring, how that may be driving racial disparities in high PSA prostate cancer in particular–including an increased understanding of the intersection of social factors of racism, social isolation, social stressors, and specific neighborhood factors—and whether such racial disparities in high PSA prostate cancer are associated with worse survival outcomes after controlling for stage and GS.

Increased serum PSA, an androgen-regulated glycoprotein molecule involved in the liquefaction of seminal fluid, is a prognostic factor of prostate cancer risk (34–36). Higher PSA among Black men relative to White men has been under investigation for decades (3, 5). Some have speculated that higher PSA levels among Black men in comparison to NHW men at diagnosis of nonmetastatic prostate cancer may be due to higher tumor cell burden or screening detection later in the clinical course (i.e., differential disease courses) (37). Tumor volume, inflammation, number, or cores positive and percentage of the higher versus lower GS components are reasons why patients could have the same stage and GS but a higher risk profile prostate cancer. Other nonmalignant clinical correlates of increased serum PSA include increasing age, larger prostate volume, infection or trauma to the prostate, and medical procedures that interfere with the prostate gland (38–42). It is therefore unclear whether high PSA among Black men compared with NHW men at diagnosis in our study is due to differences in underlying tumor biology, PSA expression, or racial differences in PSA-based screening.

There are biological differences in high PSA at prostate cancer diagnosis, which warrants further study because the majority of genetic research on prostate cancer to date has been conducted among White men. Recent work has indicated that certain *Kallikrein* polymorphisms are associated with PSA levels in Black men but not NHW men (43). Additional studies are necessary to elucidate mechanisms for biological differences in prostate cancer risk profiles observed between Black men and NHW men that are due to genetic ancestry versus social factors. Moreover, biological differences may be due underlying germline genetics or epigenetic expression in response to the embedding of racism. An example of one such study that considers the full breadth of factors that may contribute to racial disparities in prostate cancer outcomes is our national, multicenter study; Research on Prostate Cancer in Men of African Ancestry: Defining the Roles of Genetics, Tumor Markers and Social Stress (RESPOND), currently in the field. In RESPOND, we are conducting the largest coordinated research effort to date to study multilevel determinants of enduring racial disparities in prostate cancer among US Black men. We conceptualize increased risk of aggressive prostate cancer and prostate cancer mortality among Black men in the United States as a combination of underlying germline genetics and the experience of racism through individual- and neighborhood-level social stressors across the lifecourse that “get under the skin” to cause biological vulnerability in somatic profiles, tumor inflammation, and other potential mechanisms.

Disparities in PSA-based screening across populations may also contribute to high PSA among Black men at prostate cancer diagnosis. Although best available crude prevalence estimates for PSA screening are comparable between Black men and NHW men of age 40+ years in California (44, 45), these estimates may not accurately reflect PSA screening prevalence for more recent years included in our study, or relevant PSA screening behavior. Multiple PSA tests need to occur to detect prostate cancer early and it is unknown how many PSA tests Black men receive over time relative to NHW men. In our study, delayed diagnosis among Black men relative to NHW men resulting from differential longitudinal screening frequencies may have had a stronger impact on rates of high PSA at prostate cancer diagnosis than on high GS at diagnosis since prostate cancer among Black men tend to produce more PSA per tumor volume. Higher GS is generally considered to be less susceptible to early detection. We performed sensitivity analyses to examine whether Black-NHW disparities in high risk profile prostate cancer were driven by changes in screening behavior following the 2012 United States Preventives Services Task Force (USPSTF) recommendation that clinicians should not screen men who do not express a preference for screening (46). We observed fewer men with prostate cancer and proportionally more advanced prostate cancer diagnosed in 2013 to 2017 than in preceding years for both racial groups, but adjustment for a period effect of year of diagnosis 2004 to 2012 versus 2013 to 2017 did not result in changes in Black-NHW odds ratio estimates for PSA, GS, stage or prostate cancer risk of more than 1% (Supplementary Table S5). These suggest that our main findings were likely not impacted by changes in screening behavior following the 2012 USPSTF recommendations.

Our study is strengthened by the legal mandate in California for routine collection of tumor and patient characteristics on all persons with cancer in California. As part of the SEER registry program, component registries of the CCR meets stringent standards for quality, timeliness, and completeness. Hence, our study is less prone to reporting and selection biases than studies within specific healthcare systems or patient populations. We utilized composite indices for measuring nSES that did not require patient report, which enabled us to provide evidence that nSES is a primary contributor to Black-NHW disparities in advanced prostate cancer.

Our study was subject to limitations common to cancer registry-based analyses including lack of individual-level data on SES, family history of cancer, and lifestyle factors. We were unable to control for obesity, which is positively associated with GS (47), negatively associated with PSA (48), and disproportionately high in California for Black men relative to NHW men (e.g., 35.4% vs. 25.2%, respectively, in the California Health Interview Survey; ref. 49). Another limitation of SEER data is inability to identify whether men were diagnosed with prostate cancer following routine screening or based on a symptomatic indication. An additional limitation of cancer registry data is lack of information regarding prior residences or length at residence, which may be related to prostate cancer risk profiles. We also lacked data on tumor volume, prostate size, and number and involvement of biopsy cores. Furthermore, our single PSA test result lacked potentially important information such as PSA kinetics (PSA velocity and doubling time) and free-to-total PSA ratio; potentially important predictors of prostate cancer risk profile (35, 36, 50). In addition, we observed some indication of nonrandom missingness in prostate cancer risk profile outcome variables. The largest proportion of missing prostate cancer risk profile data were observed among men with unknown or missing insurance status. However, men with missing or unknown insurance status comprised <5% of the overall sample. A recent study examining potential exclusion bias due to missing data when grouping prostate cancer cases using this D’Amico risk stratification in SEER data, found that tumor characteristics among men with missing prostate cancer risk profile data were similar to those with complete data for risk profile (51). Our observation of <1% differences in the ORs between our models with and without multiple imputations provided evidence that our findings were likely not biased due to missing data.

Our findings suggest that racial disparities in high-risk prostate cancer among Black men relative to NHW men may be influenced in part by differences in nSES. These findings are consistent with previous findings of high prostate cancer risk profiles for men in the lowest nSES quintile (2) and worse prostate cancer survival among Black men than White men, which was attenuated by adjustment for nSES (23). Our findings that marital status contributes to racial disparities in high-risk prostate cancer among Black men relative to White men is consistent with findings that marital status is an independent and strong predictor of prostate cancer survival, and a moderator of racial disparities therein (7, 24). We interpret our findings for nSES and marital status in light of emerging evidence that prostate cancer develops through complex interactions at the biological, individual, and social levels (10, 23, 52, 53). Further work is necessary to elucidate potentially relevant adverse exposures among Black men residing in low nSES neighborhoods, such as perceived racism and social stress, how such factors may contribute to high-risk prostate cancer profiles. Furthermore, the association between high-risk prostate cancer at diagnosis and unmarried status warrants additional investigation, possibly to inform social support resources for health disparities populations.

## Authors' Disclosures

D.J. Press reports other support from Genentech A Member of the Roche Group outside the submitted work. S. Shariff-Marco reports grants from NCI during the conduct of the study. D.Y. Lichtensztajn reports grants from NCI during the conduct of the study. P.P. Inamdar reports grants from NCI during the conduct of the study. M.C. DeRouen reports grants from UCSF during the conduct of the study. A.S. Hamilton reports grants from NCI during the conduct of the study. K. Lin reports grants from NCI during the conduct of the study. C.A. Haiman reports grants from NCI during the conduct of the study. S.L. Gomez reports grants from NIH during the conduct of the study. No disclosures were reported by the other authors.
